# Supplementary material for: Patient experiences of diabetes and hypertension care during an evolving humanitarian crisis in Lebanon: A qualitative study
Source: PLOS Glob Public Health. 2023 Dec 6;3(12):e0001383. doi: 10.1371/journal.pgph.0001383 (PMC10699589; doi:10.1371/journal.pgph.0001383)
Supplement: S1 Checklist — (DOCX) [file pgph.0001383.s001.docx]

STROBE Statement—checklist of items that should be included in reports of observational studies: Manuscript: PGPH-D-22-01619

*Note: please see also separate COREQ (Consolidated Criteria for Reporting Qualitative Studies) checklist*

|  | Item No. | Recommendation | Page  No. | Relevant text from manuscript |
| --- | --- | --- | --- | --- |
| **Title and abstract** | 1 | (*a*) Indicate the study’s design with a commonly used term in the title or the abstract | 1 | ‘a qualitative study’ |
|  |  | (*b*) Provide in the abstract an informative and balanced summary of what was done and what was found | 2 | ‘this study explored experiences of displaced Syrian and vulnerable Lebanese patients receiving care for hypertension and/or diabetes at four health facilities supported by humanitarian organisations in Lebanon. We conducted in-depth, semi-structured qualitative interviews with a purposive sample of patients (n=18) and their informal care-givers (n=10). Data were analysed thematically using both deductive and inductive approaches. Both Syrian and Lebanese patients reported interrupted pathways of care. We identified three typologies of patient experience at the time of interview; (1) managing adequately from the patient’s perspective; (2) fragile management and (3) unable to manage their condition(s) adequately, with the majority falling into typologies 2 and 3. Patients and their families recognised the importance of maintaining continuity of care and self-management, but experienced substantial challenges due to changing availability and cost of medications and services, and decreasing economic resources during a period of national crises. Family support underpinned patient’s response to challenges. Navigating the changing care landscape was a significant burden for patients and their families. Interactions were identified between mental health and NCD management. This study suggests that patients experienced disrupted, non-linear pathways in maintaining care for hypertension and diabetes in a humanitarian setting, and family support networks were key in absorbing treatment burden and sustaining NCD management.’ |
| Introduction | | | |  |
| Background/rationale | 2 | Explain the scientific background and rationale for the investigation being reported | Pages 2-3  Lines 56-75  Page 3  Lines 83-85 | ‘Patients with noncommunicable diseases (NCDs) who are forcibly displaced in humanitarian crises face challenges of flight and displacement as well as the need for long-term management of health conditions and the attendant psychosocial impacts (1-3). Monitoring and continuous treatment of existing NCDs is essential to prevent complications, preserve quality of life, and avoid economic burden to the household and health system, but this is especially difficult to maintain in crisis situations (2, 4). Both the global burden of NCDs and the numbers of people forcibly displaced are increasing, and both disproportionately affect populations in low and middle income countries (LMIC) , intensifying challenges for health systems (5-7). In response, humanitarian health care models, which traditionally focused on acute, episodic care, are being adapted to include care for existing NCDs (8, 9). This mirrors the wider health system ‘paradigm shift’ in care for chronic conditions advocated by the World Health Organisation (WHO) in 2002 (10).  In this emerging field, attention at policy and practice levels has been on service provision, positioned from the perspectives of actors who are establishing approaches to embed NCD care in humanitarian models (1, 2, 7, 11-14). There has been less focus on patient’s experiences of receiving NCD care during humanitarian crises, with the exception of recent research in Lebanon addressing access to primary health care services (14-16). Our research seeks to address this gap, with a focus on the continuum of care for patients who are accessing services. We draw on the concept of continuity of care as defined by WHO: ‘the extent to which a series of discrete health care events is experienced by people as coherent and interconnected over time and consistent with their health needs and preferences’  ‘The partnership identified hypertension and diabetes as focal conditions due to their high disease burden in the study context (19, 20) and globally (21), frequent co-existence and amenability to a primary level public health approach for management and prevention of complications’ |
| Objectives | 3 | State specific objectives, including any prespecified hypotheses | Page 3 lines 75-77 | (hypothesis not applicable for this exploratory qualitative study)  A specific aim is stated, as appropriate for this study type: ‘The aim of this paper is to explore the experiences of patients receiving care for hypertension and/or diabetes at four health facilities supported by humanitarian organisations in Lebanon during 2020-21’ |
| Methods | | | | In addition please see separate COREQ (Consolidated Criteria for Reporting Qualitative Studies) checklist |
| Study design | 4 | Present key elements of study design early in the paper | Page 4 Lines 122-124 | ‘We conducted in depth, semi-structured qualitative interviews with patients (n=18) and informal caregivers (i.e family members/neighbours/friends who support patients with their NCD condition) (n=10).’  (further details re sampling lines 124-134) |
| Setting | 5 | Describe the setting, locations, and relevant dates, including periods of recruitment, exposure, follow-up, and data collection | Page 4 – 5 Lines 114-149 | **Setting and locations**: lines 114-120: ‘The study was set in four health facilities providing NCD services, supported by P4C partners and serving urban catchment areas with substantial refugee populations (estimated 30-45% of total catchment population) in North Lebanon and Mount Lebanon governorates. Two were primary-level dispensaries, one a primary health care centre, and one a division of a governmental tertiary hospital providing emergency inpatient care. All provided low cost or free-of-charge health services to Syrian refugee and vulnerable Lebanese patients with previously diagnosed hypertension or diabetes.’  **Location of interview**: (via telephone) lines 134-136 ‘Following COVID-19 social distancing guidance, patients were contacted by telephone for invitation to participate, using an oral recruitment script, and subsequently for telephone interview from a private university office.’  **Period of recruitment and data collection**: September 2020 - February 2021  Exposure and follow-up are not applicable for this study. |
| Participants | 6 | (*a*) *Cohort study*—Give the eligibility criteria, and the sources and methods of selection of participants. Describe methods of follow-up  *Case-control study*—Give the eligibility criteria, and the sources and methods of case ascertainment and control selection. Give the rationale for the choice of cases and controls  ***Cross-sectional study***—Give the eligibility criteria, and the sources and methods of selection of participants | Page 4-5 | **Eligibility criteria**: ‘Patients aged 18 years or over, diagnosed with hypertension/diabetes (HTN/DM) at least 5 years ago, and receiving care at one of the four study health facilities were eligible for interview’  **Sources and methods of selection of participants**: ‘receiving care at one of the four study health facilities’….. ‘Patients were purposively sampled to represent a range of demographic (gender, age, nationality) and condition-related (hypertension and/or diabetes, with/without complications, such as previous heart attack, stroke, diabetes-related amputation or visual impairment) groups, and then randomly selected from within these groups. Sampling criteria were identified by the humanitarian study partners in consultation with facility management. The sample was broadly representative of the HTN/DM patient population at each facility, weighted to ensure representation of those with known complications, in order to include experiences at multiple health system levels’ |
|  |  | (*b*) *Cohort study*—For matched studies, give matching criteria and number of exposed and unexposed  *Case-control study*—For matched studies, give matching criteria and the number of controls per case |  | Not applicable (Cross-sectional study) |
| Variables | 7 | Clearly define all outcomes, exposures, predictors, potential confounders, and effect modifiers. Give diagnostic criteria, if applicable |  | Not applicable – qualitative study |
| Data sources/ measurement | 8* | For each variable of interest, give sources of data and details of methods of assessment (measurement). Describe comparability of assessment methods if there is more than one group |  | Not applicable – qualitative study |
| Bias | 9 | Describe any efforts to address potential sources of bias | P 4-5 lines 127-130  P 19 lines 593-597 | Addressed through purposive sampling: ‘Patients were purposively sampled to represent a range of demographic (gender, age, nationality) and condition-related (hypertension and/or diabetes, with/without complications, such as previous heart attack, stroke, diabetes-related amputation or visual impairment) groups, and then randomly selected from within these groups  Addressed through discussion of limitations: ‘The scope of the study population was limited by the need to conduct telephone interviews, excluding patients without access to a telephone who may experience greater challenges in maintaining care. By focusing on experiences of continuity of care among patients receiving care, those who were not diagnosed or not accessing services were outside the scope of the study.’ |
| Study size | 10 | Explain how the study size was arrived at | P 4 lines 124-5 | ‘Sample size was guided by experience from previous research with parents and caregivers and by project resources’ |

Continued on next page

| Quantitative variables | 11 | Explain how quantitative variables were handled in the analyses. If applicable, describe which groupings were chosen and why |  | Not applicable – qualitative study  **Please see separate COREQ (Consolidated Criteria for Reporting Qualitative Studies) checklist** |
| --- | --- | --- | --- | --- |
| Statistical methods | 12 | (*a*) Describe all statistical methods, including those used to control for confounding |  | Not applicable - qualitative study |
|  |  | (*b*) Describe any methods used to examine subgroups and interactions |  |  |
|  |  | (*c*) Explain how missing data were addressed |  |  |
|  |  | (*d*) *Cohort study*—If applicable, explain how loss to follow-up was addressed  *Case-control study*—If applicable, explain how matching of cases and controls was addressed  *Cross-sectional study*—If applicable, describe analytical methods taking account of sampling strategy |  |  |
|  |  | (*e*) Describe any sensitivity analyses |  |  |
| Results | | | | |
| Participants | 13* | (a) Report numbers of individuals at each stage of study—eg numbers potentially eligible, examined for eligibility, confirmed eligible, included in the study, completing follow-up, and analysed | Page 6 line 174 | ‘We interviewed 18 patients and 10 caregivers’  (see also (b) below) |
|  |  | (b) Give reasons for non-participation at each stage | Page 6 line 174-5  184-5 | ‘Eleven patients initially identified as eligible did not participate (three did not meet criteria, seven were not contactable, one declined).’  ‘Caregivers were identified by twelve patients, and ten were contactable and willing to participate in interviews’ |
|  |  | (c) Consider use of a flow diagram |  | Not applicable – small qualitative study |
| Descriptive data | 14* | (a) Give characteristics of study participants (eg demographic, clinical, social) and information on exposures and potential confounders | Page 6 line 175-83  Lines 185-192 | **Characteristics of study participants:**  ‘Patients were aged between 29-73 years, with two patients aged under 40 years. Equal numbers (n=9) were male and female. Ten patients were Syrian and eight were Lebanese. The majority of patients (n=12) were married, and three were single, two divorced and one widowed. Eight patients had been diagnosed with diabetes, four with hypertension (HTN), and six with both hypertension and diabetes (HTN&DM). One patient had been diagnosed three years prior to being interviewed; the remaining 17 had been diagnosed 5-20 years previously. Ten patients reported having experienced complications (two patients with hypertension; three comorbid patients, five patients with diabetes).’  ‘Caregivers were aged 27-65 years, with five aged under 40 years. Equal numbers were male and female. Seven were Syrian and three were Lebanese. All were family members of patients, including spouses (n=4), children (n=3), one parent, sibling and cousin. Two caregivers reported having hypertension, and one of these had experienced complications.  One-third of respondents reported being illiterate and one-third had primary school education only. Two-thirds of participants were unemployed and 2 of 28 participants were in formal employment. All participants reported worsening financial situations due to the economic crisis in Lebanon and COVID-19 pandemic.’  **Exposures and confounders**: Not applicable |
|  |  | (b) Indicate number of participants with missing data for each variable of interest |  | Not applicable - qualitative study |
|  |  | (c) *Cohort study*—Summarise follow-up time (eg, average and total amount) |  |  |
| Outcome data | 15* | *Cohort study*—Report numbers of outcome events or summary measures over time |  |  |
|  |  | *Case-control study—*Report numbers in each exposure category, or summary measures of exposure |  |  |
|  |  | *Cross-sectional study—*Report numbers of outcome events or summary measures |  | Not applicable - qualitative study |
| Main results | 16 | (*a*) Give unadjusted estimates and, if applicable, confounder-adjusted estimates and their precision (eg, 95% confidence interval). Make clear which confounders were adjusted for and why they were included |  | Not applicable - qualitative study  **Please see separate COREQ (Consolidated Criteria for Reporting Qualitative Studies) checklist** |
|  |  | (*b*) Report category boundaries when continuous variables were categorized |  |  |
|  |  | (*c*) If relevant, consider translating estimates of relative risk into absolute risk for a meaningful time period |  |  |

Continued on next page

| Other analyses | 17 | Report other analyses done—eg analyses of subgroups and interactions, and sensitivity analyses |  |  |
| --- | --- | --- | --- | --- |
| Discussion | | | | |
| Key results | 18 | Summarise key results with reference to study objectives | P16-17 lines 513-525 | **Please see separate COREQ (Consolidated Criteria for Reporting Qualitative Studies) checklist**  Summary of findings |
| Limitations | 19 | Discuss limitations of the study, taking into account sources of potential bias or imprecision. Discuss both direction and magnitude of any potential bias | Page 19 lines 593-7 | ‘The scope of the study population was limited by the need to conduct telephone interviews, excluding patients without access to a telephone who may experience greater challenges in maintaining care. By focusing on experiences of continuity of care among patients receiving care, those who were not diagnosed or not accessing services were outside the scope of the study’ |
| Interpretation | 20 | Give a cautious overall interpretation of results considering objectives, limitations, multiplicity of analyses, results from similar studies, and other relevant evidence | Page 16 -18  Page 17  Page 18 | Interpretation is embedded throughout the discussion section, comparing findings with existing evidence and theoretical frameworks, for example:  ‘This non-linearity resonates with findings of a recent review of pathways for hypertension care and control in LMICs, which suggests that patient pathways ‘are best characterised as continual cycles of entry and re-entry into the system’(50).’  ‘The delegation of ‘work’ from a health system to patients with chronic conditions and their social networks has been described in relation to the concept of ‘burden of treatment’, which recognises the workload of healthcare and its impact on patient functioning and well-being (55, 56). For participants in this study, a substantial additional ‘workload’ was generated by the changing availability and cost of key elements of care, in parallel with patients’ decreasing ability to afford them.’ |
| Generalisability | 21 | Discuss the generalisability (external validity) of the study results |  | This is not a reporting requirement for qualitative studies (see COREQ checklist) and we do not make claims to generalisability, however recommendations based on our findings are made at specific levels (lines 602-697 page 19-120) for example for the Lebanese Ministry of Public Health, and for implementing partners, which may be implemented at different levels (in national setting in Lebanon, or through their international programmes). |
| Other information | |  | | |
| Funding | 22 | Give the source of funding and the role of the funders for the present study and, if applicable, for the original study on which the present article is based |  | This is given in the funding statement uploaded separately from the main manuscript as directed by PLoS GPH:  ‘This work was supported by a grant from the Novo Nordisk A/S, Global Access to Care Department to the London School of Hygiene and Tropical Medicine (EA, KB, PP, BR),  as part of the Partnering for Change collaboration between the International Committeeof the Red Cross (ICRC), Danish Red Cross (DRC) and Novo Nordisk’  ‘The funder had no role in study design, data collection and analysis, decision to publish, or preparation of the manuscript’ |

*Give information separately for cases and controls in case-control studies and, if applicable, for exposed and unexposed groups in cohort and cross-sectional studies.

**Note:** An Explanation and Elaboration article discusses each checklist item and gives methodological background and published examples of transparent reporting. The STROBE checklist is best used in conjunction with this article (freely available on the Web sites of PLoS Medicine at http://www.plosmedicine.org/, Annals of Internal Medicine at http://www.annals.org/, and Epidemiology at http://www.epidem.com/). Information on the STROBE Initiative is available at www.strobe-statement.org.
