# Supplementary material for: Patient experiences of diabetes and hypertension care during an evolving humanitarian crisis in Lebanon: A qualitative study
Source: PLOS Glob Public Health. 2023 Dec 6;3(12):e0001383. doi: 10.1371/journal.pgph.0001383 (PMC10699589; doi:10.1371/journal.pgph.0001383)
Supplement: S2 Checklist — (PDF) [file pgph.0001383.s002.pdf]

| **Consolidated criteria for reporting qualitative studies (COREQ): 32-item checklist:** [COREQ (Consolidated Criteria for Reporting Qualitative Studies) - Guidelines for Reporting Health Research: A User's Manual - Wiley Online Library]<br>Study Title: Patient experiences of diabetes and hypertension care during an evolving humanitarian crisis in Lebanon: a qualitative study | | | |
|---|---|---|---|
| **No.** | **Item** | **Guide questions/description** | **Page number/paragraph and section of manuscript which reports the information that meets the criteria of the checklist** |
| **Domain 1: Research team and reflexivity** | | | |
| **Personal Characteristics** | | | |
| 1. | Interviewer/facilitator | *Which author/s conducted the interview or focus group?* | Page 5 line 146-9, Methods section |
| 2. | Credentials | *What were the researcher's credentials? E.g. PhD, MD* | Page 5 line 148-9 (credentials of research assistant conducting interviews), Methods section |
| 3. | Occupation | *What was their occupation at the time of the study?* | Page 5 line 148, Methods section |
| 4. | Gender | *Was the researcher male or female?* | Page 5 line 148, Methods section |
| 5. | Experience and training | *What experience or training did the researcher have?* | Page 5 line 148-9, Methods section |
| **Relationship with participants** | | | |
| 6. | Relationship established | *Was a relationship established prior to study commencement?* | Page 4 line 127-136, Methods section |
| 7. | Participant knowledge of the interviewer | *What did the participants know about the researcher? e.g. personal goals, reasons for doing the research* | Page 5 line 135-140, Methods section<br>Supplementary File: Topic Guide Page 1 |
| 8. | Interviewer characteristics | *What characteristics were reported about the interviewer/facilitator? e.g. Bias, assumptions, reasons and interests in the research topic* | Page 5 line 148-9, Methods section |
| **Domain 2: study design** | | | |
| **Theoretical framework** | | | |
| 9. | Methodological orientation and Theory | *What methodological orientation was stated to underpin the study? e.g. grounded* | Page 5 line 158-62, Methods section |

| | | | |
|---|---|---|---|
| | | *theory, discourse analysis, ethnography, phenomenology, content analysis* | |
| **Participant selection** | | | |
| 10. | Sampling | *How were participants selected? e.g. purposive, convenience, consecutive, snowball* | Pages 4 lines 124-134, Methods section |
| 11. | Method of approach | *How were participants approached? e.g. face-to-face, telephone, mail, email* | Page 5 line 134, Methods section |
| 12. | Sample size | *How many participants were in the study?* | Page 4 lines 122-124, Methods section<br>Page 6 line 174, Results section |
| 13. | Non-participation | *How many people refused to participate or dropped out? Reasons?* | Page 6 line 174-5, 184, Results section |
| **Setting** | | | |
| 14. | Setting of data collection | *Where was the data collected? e.g. home, clinic, workplace* | Page 5 lines 135-6, Methods section |
| 15. | Presence of non-participants | *Was anyone else present besides the participants and researchers?* | Page 5 lines 135-6, Methods section |
| 16. | Description of sample | *What are the important characteristics of the sample? e.g. demographic data, date* | Page 6 lines 175-192, Results section |
| **Data collection** | | | |
| 17. | Interview guide | *Were questions, prompts, guides provided by the authors? Was it pilot tested?* | Page 6 lines 144-6, Methods section<br>Supplementary File: Topic Guide |
| 18. | Repeat interviews | *Were repeat interviews carried out? If yes, how many?* | N/A - No, it was not appropriate to conduct repeat interviews for this study design. |
| 19. | Audio/visual recording | *Did the research use audio or visual recording to collect the data?* | Page 5, line 151, Methods section |
| 20. | Field notes | *Were field notes made during and/or after the interview or focus group?* | N/A - No additional field notes were made beyond researcher's notes during interviews (notes of key responses in case recording failed). As this is not an observational/ethnographic study design we would not expect to report on or draw on field notes. If they had been taken we would report accordingly but do not feel it is appropriate to state in the manuscript that they were not taken. |

| | | | |
|---|---|---|---|
| 21. | Duration | *What was the duration of the interviews or focus group?* | Page 5 line 147, Methods section |
| 22. | Data saturation | *Was data saturation discussed?* | No - sample size was guided by experience from previous research with patients and caregivers and by project resources, although data saturation was also a consideration |
| 23. | Transcripts returned | *Were transcripts returned to participants for comment and/or correction?* | N/A - No, we did not judge that this was necessary use of participant's time and project resources for this study topic, design and participant group. |
| **Domain 3: analysis and findings** | | | |
| **Data analysis** | | | |
| 24. | Number of data coders | *How many data coders coded the data?* | Page 5 lines 159-60, Methods section |
| 25. | Description of the coding tree | *Did authors provide a description of the coding tree?* | No – the overall coding framework reflected the research priorities of the wider project, and this paper presents a subset of these results which cut across different sections and levels of the overall coding framework |
| 26. | Derivation of themes | *Were themes identified in advance or derived from the data?* | Page 5 lines 158-62, Methods section |
| 27. | Software | *What software, if applicable, was used to manage the data?* | Page 6 line 165, Methods section |
| 28. | Participant checking | *Did participants provide feedback on the findings?* | No - for patients and care-givers we did not judge that this was necessary use of participant's time and project resources for this study topic, design and participant group, given that social distancing requirements meant that any feedback processes would need to be via telephone/virtual. Findings were shared with humanitarian partner country and HQ level teams who provided feedback. |
| **Reporting** | | | |
| 29. | Quotations presented | *Were participant quotations presented to illustrate the themes / findings? Was each quotation identified? e.g. participant number* | Quotations are presented to illustrate themes throughout the results section (pages 8-17), identified by participant number, condition and nationality, Results section |
| 30. | Data and findings consistent | *Was there consistency between the data presented and the findings?* | The findings were derived from the data, and data is embedded in presentation of each theme (pages 6-16), Results section |

| | | | |
|---|---|---|---|
| 31. | Clarity of major themes | *Were major themes clearly presented in the findings?* | Reporting of findings (pages 6-16) is structured by theme, as summarised in lines 196-202 page 7, Results section |
| 32. | Clarity of minor themes | *Is there a description of diverse cases or discussion of minor themes?* | Results section (pages 6-16) – cases are presented by typology which describes diversity of cases, eg lines 314-317 page 10.<br>Minor themes are presented together with major themes, for example in discussion of mental health (p15-16) description of the main theme (lines 477-8) is followed by discussion of sub-themes (lines 481-93). Attention is paid to (1) emerging themes which were not anticipated in the theoretical framework, for example importance of scope of family support (pages 15, lines 461-75) and (2) diverse cases (e.g. patient A typology 1, page 10, lines 314-17). |
