# Supplementary material for: Patient experiences of diabetes and hypertension care during an evolving humanitarian crisis in Lebanon: A qualitative study
Source: PLOS Glob Public Health. 2023 Dec 6;3(12):e0001383. doi: 10.1371/journal.pgph.0001383 (PMC10699589; doi:10.1371/journal.pgph.0001383)
Supplement: S1 Text — (DOCX) [file pgph.0001383.s003.docx]

Manuscript: *Patient experiences of diabetes and hypertension care during an evolving humanitarian crisis in Lebanon: a qualitative study*

**Semi-structured Patient or Carer Interview Guide: English Language Version**

**INSTRUCTIONS TO INTERVIEWERS**

1. Start with the open questions listed below.
2. Probes are follow-up questions to be used as needed to initiate discussion or elicit further contributions. If probing is not necessary to initiate discussion or elicit further contributions, probes should not be asked. Probes in this guide are listed below the main questions and are italicised.
3. Global Probes can be used any time at the facilitator’s discretion to generate more complete answers or further reflection:

- *Please say more about that.*
- *Please give me an example of that.*
- *Tell me more about your thinking about that.*
- *What led up to that?*
- *What happened after that?*

**Information to give to respondents before conducting the interview:**

[Following COVID-19 Social Distancing amendment complete separate Verbal Informed Consent for Patients]

1. Inform respondents that the aim of this evaluation is to understand the health needs of people with or without complications of diabetes and hypertension, and the current functioning and quality of the services and systems available to address these needs.
2. The objective is to understand what health care activities were implemented, and to describe key lessons learned – both positive and negative – for the purposes of informing future activities. We want to understand what works well and should be replicated in other facilities and settings, and what to modify and avoid.
3. Inform respondents that no names or personal identifiers will be included in transcripts or reports.
4. We estimate that the interview will take approximately one to one and a half hours to complete.

**Interview with:** *TICK ALL THAT APPLY*

- Patient with hypertensive complication – stroke
- Patient with hypertensive complication – heart attack
- Patient with diabetic complication - amputation
- Patient with diabetic complication – retinopathy leading to visual impairment
- Patient with diabetic complication – renal failure (eGFR, 30/dialysis)
- Patient with hypertension and no complications
- Patient with diabetes and no complications
- Other (specify) _____________________________

__________________ (Interviewer’s initials)

_____/ _____/ _____ (Date)

Data about the participant

Please fill out the following details for each interview:

| Age | 18-24  25-34  35-44  More than 44 |
| --- | --- |
| Gender | Female  Male |
| Nationality | Lebanese  Syrian |
| Education | Illiterate  Intermediate  High school  Higher Education  Technical |
| Employment Status |  |
| Marital Status |  |
| Centre visited | [x]  [x]  [x] |
| Diagnosis | Diabetes  Hypertension  Both |
| Duration since diagnosis |  |
| Complications |  |

| Key area | Themes | Question |
| --- | --- | --- |
| Introduction | Study aim and agencies involved  Why invited to participate  Consent & any questions? | [Refer to: Verbal Informed Consent for Patients] |
| Participant Background | Getting to know each other & building rapport | Could you tell us a bit about yourself?   - *Prompt: e.g. Ask about age, where they currently reside, their family circumstances and if appropriate, current work/study. How long living in Lebanon, with family or not? Have you moved within Lebanon? Where and when?* |
| Patient Pathway - Diagnosis | Diagnosis  Initial care seeking and supports  Prior knowledge and knowledge in community  (Limit this to DM, HTN, CVD) | What medical condition are you (or your family member) attending care for in Lebanon? (self-care, pharmacy, community groups)   - *Prompt – If more than one condition or complication, ask about each separately*   What made you (or your family member) look for help for this condition and where did you go for help?   - *Prompts – feeling unwell? What symptoms? When did symptoms start e.g.in Lebanon or in home country?* - *Prompted by family member or friend, community worker, messaging via radio, television, internet?* - *How long ago did you seek help, from where? (4Ws, what links exist between different levels/sites of care?).If yes, did they refer you to other health care centers?* - *Was there any delay in seeking care and, if yes, what was the reason? (cost, distance, knowledge, social constraints, political unrest, corona crisis)* - How did you feel when you were first diagnosed? (*anxiety, worry, fear, acceptance, anger etc. )*   What supports and information did you (or your family member) initially receive after you/they were first diagnosed?   - a) at the health facility and b) in the community. - *Prompts –? Kind of information, by whom, how (verbal, written material, web resources, support or community group)* - *Useful/appropriate/comprehensible?* - *Other sources of information (e.g. internet, family, community members, neighbours, pharmacist, TV etc.)? Are they useful?*   What did you know about your (your family member’s) condition before you (or your family member) were/was diagnosed?   - *Prompt –* source of knowledge (incl. internet, radio, family members, community etc); - type of *knowledge : cause, prevention, treatment, prevention and treatment of complications, implications for future.*   How much do you think the broader community knows or understands about your (your family member’s) condition?   - *Prompt - among family, neighbours, friends, children; type of knowledge and sources of knowledge as above)*   Can you describe the flow of information between you and other people with the same diagnosis?   - - Have you received any advices or information from other diabetic/HTN patients?   - Have you tried to help other people with similar diagnosis? |
| Patient Pathway – Current care | Current care and support  Community based care  Access | What services or supports do you (or your family member) currently use to manage your condition? *[possible COVID19 implications here]*   - *Prompt - Type of facility and what offered – 4 Ws (who, what, where, how);* - *Medications prescribed (Specify to which condition) (i.e. indication not name); other source(s), cost, management of medication regime (of insulin if relevant) adherence and support, gaps in supply (what do you do if you are short on medication?). Where do you get your medications from? Do you change your insulin dosage? Who injects the insulin?* - *Equipment provided; (e.g. Glucometer, lancets, syringes, needles, BP cuff, exercise equipment)/ appropriate? Any difficulties with accessing or using these?* - *Investigations? (e.g. Blood tests, foot check, eye check, ECG; any access difficulties?* - *Healthy living education or intervention (note will be focused on in a later question)(Diet, exercise, smoking, dietary support, awareness sessions..)* - *Referral services (secondary/tertiary care, MHPSS, physio or rehab, protection services, links to other refugee services?)* - *If has lived elsewhere in Lebanon, how do your current services compare to previous services?* - *How do your current services compare to services prior to the coronavirus crisis?* - *Current support from friends and family?*   Are you (your family member) accessing any community based care at the moment? (i.e pharmacy)   - *Community based groups, organisations, volunteers, formal/informal, non DM/HTN related? Are there any groups you are involved in?*   How easy or difficult is it to access these services?   - If not already covered, discuss what makes it easy/ difficult? *(cost, availability, distance/ transport, social constraints, coronavirus crisis)* |
|  | Daily routine  Healthy living  Barriers and facilitators to self-care  Wellbeing  Complications | Could you tell me about your (or your family member’s) daily routine around managing your/their condition? *[possible COVID19 implications here]*   - *Daily routine, nutrition, physical exercise [reduced movement due to political unrest/corona crisis lockdown], impact on daily living, family life, work life; functionality re activities of daily living (washing, dressing, household tasks)/work/school* - *Symptoms (any pain)? Effect on quality of life?* - *Challenges (knowledge, adherence meds/ lifestyle, stigma, worries, beliefs about the illness; financial impact)* - *What helps you to look after your illness? Current supports / additional supports needed? (Family, friends, community-based, financial, psychological supports, access to healthy food /exercise options, tailored advice, rehab/ physio/palliative care)*   What is the main worry for you (or your family member) around managing your/ their illness?   - *Prompt - Does the condition affect your mood and sense of well-being?* - *Cause you to feel upset or cause stress? Sources of support if you feel upset or stress/ worried/ anxious? Do you talk to anyone? (Family, friends, neighbours, community, CHW, HCP, religion, books, Internet).* - *Specific services or supports available for people who are distressed? (Counselling, psychology, psychiatry; what supports/services, where, by whom, how to access?). Supports offered by health care worker/CHW?*   **A. Could you tell me a bit more about the complication (s) of your (or your family member’s) hypertension/diabetes (s) (neuropathy, lack of sensation in your peripheries, heart procedures? Angiograms, narrowing of arteries)**   - Prompt - from among heart attack, stroke, renal failure, visual impairment, heart failure and/or amputation etc. - *Re complication: what happened, when, where, help seeking, if delay & reasons; beliefs about why complication happened, knowledge re prevention and treatment of complications; specific services, challenges, what supports would help.*   **OR B. Your condition is very well controlled and you have not experienced any complications. Could you tell me about how you have managed your condition so well?**   - *Prompt - if not covered: help seeking, any delay & reasons; beliefs re lack of complications, knowledge re complications; general beliefs about illness / medications, how currently managing day-to-day, family or community support, what services available, what challenges facing, what supports would help?)* |
| Quality/ Patient-centeredness of current care | Access and coverage  Experience, respect and dignity  Communication  User focus  Quality of infrastructure | If attending more than one service, ask in relation to each and compared to services accessed elsewhere in Lebanon.  What has your (or your family member’s) experience of DM/HTN services/care/support been like?   - *Prompt - if attending more than one service/location, ask this about each* - *How did you feel about the care/treatment you were given? (Satisfied, trust in service and providers; if unsatisfied: probe for changes desired]*   How did you feel you (or your family member) were/was treated by staff?   - *Prompt – respect, dignity, privacy, non-discrimination, autonomy, confidentiality* - *Clear explanation, good communication e.g. time to ask questions, questions satisfactorily answered, speaks slowly, verifies what patient understood, accessible language, use of mother tongue* - *If you had questions about your treatment or an emergency do you have a point of contact?*   What do you think about the quality of services you are currently receiving for your HTN/DM?   - *Prompt - sufficient, acceptable – type, amount, location, quality, trust, appropriate to you/family member and refugee status (if appropriate) – meeting expectations?* - Perceived competence, numbers, and training of providers, medicine, and equipment. - Adequate referral services - Physical access (distance, transport cost /availability, physical/cultural/security impediments); Language; Accommodation (appointment system, waiting times, flexible opening hours, walk in if urgent) - Cost (any payments including outside of facility; how paid; affordability vs. household income, financial choices made)   Can you tell me about your experience of being involved in decisions about your (your family member’s) health care or treatment?   - *Prompt – patient voice, values, asked for input, asked re concerns, family included* - *Offered choice of provider, info re other types of services for condition, freedom to choose provider/service/treatment)*   *(i.e compare between primary and secondary care)*  Can you tell me about the quality of the facilities where you (your family member) are/is receiving care?   - *Prompt – privacy, confidentiality, comfort, cleanliness, quality of clinic room/ waiting room (space, seating, fresh air).* |
| Integration and continuity of current care | Integration  Continuity of care | Do you have regular contact with the health centre/hospital or health care provider you (or your family member) attend(s)?   - *Prompt –how often, appointment based? access outside appointment times? recall or reminder systems?*   **How is continuity of care assured?** *[possible COVID19 implications here]*   - *Prompts – Regular follow up: How often seen, appointment based? Access outside appointment times? recall or reminder systems? defaulter tracing, community outreach;* - *Continuity of information: registration book, patient held/clinic-based file, what data collected/ shared with other providers, what referral system is in place and how well does it function* - *Continuity of personnel: same or different clinical staff, any key continuous relationship;* - *Consistency of care between different health system sites/levels*   How do the current services support you (your family member) to manage your multiple conditions (if relevant)?   - *Probe: Adapt advice/ services to multiple symptoms/ functionality/ medications? Manage all conditions / refer? Different providers for different conditions? How do you personally manage (symptoms, treatments, providers)?* |
| Thanks and  close | Anything else to add  Questions/Thanks | Is there anything else you could suggest that would improve management of your DM/HTN for you?  Can you think of a new or innovative approach to managing your DM/ HTN?  Is there anything else you would like to add?  Do you have any additional questions for me?  Are you all still happy to have your data included for this evaluation?  **Thank you for your time.** |
