# Supplementary material for: Patient experiences of diabetes and hypertension care during an evolving humanitarian crisis in Lebanon: A qualitative study
Source: PLOS Glob Public Health. 2023 Dec 6;3(12):e0001383. doi: 10.1371/journal.pgph.0001383 (PMC10699589; doi:10.1371/journal.pgph.0001383)
Supplement: S2 Text — (DOCX) [file pgph.0001383.s004.docx]

Inclusivity in global research

PLOS’ policy on inclusivity in global research aims to improve transparency in the reporting of research performed outside of researchers’ own country or community and ensures that PLOS publications reporting global research adhere to high standards for research ethics and authorship. Authors of relevant research articles may be asked to complete the questionnaire below, which outlines ethical, cultural, and scientific considerations specific to inclusivity in global research. This questionnaire may be requested when researchers have travelled to a different country to conduct research, if research uses samples collected in another country, research with Indigenous populations or their lands, or if research is on cultural artefacts. Researchers travelling to another country solely to use laboratory equipment will not normally be required to complete the questionnaire. However, the questionnaire can be requested at the journal’s discretion for any submission – if you have been requested to complete this questionnaire by the PLOS journal you submitted to, please do so.

Please complete the questionnaire below and include this as a Supporting Information file with your manuscript. Note that if your paper is accepted for publication, this checklist will be published with your article in the supporting information files. Please ensure that you reference the checklist in the main body of your manuscript. We suggest adding a subsection ‘Inclusivity in global research’ to your Methods section and adding the following sentence: “Additional information regarding the ethical, cultural, and scientific considerations specific to inclusivity in global research is included in the Supporting Information (SX Checklist)”

The questions have been designed to be applicable to a wide range of study types, and there are subsections for both human subjects research and non-human subjects research. If any of the questions are not relevant to your research please mark them as “N/A” as appropriate.

**Ethical considerations, permits and authorship**

*This section is applicable to all research types.*

Provide details as to who granted permissions and/or consent for the study to take place in the Methods section of your manuscript. This should include the names of **all** ethics boards, governmental organizations, community leaders or other bodies that provided approval for the study. If individuals provided approval refer to these people by their role or title but do not list their name(s).

Reported on page number: Page 7

If there were any deviations from the study protocol after approval was obtained please provide details of these changes in the Methods section of your manuscript.
Did this study involve local collaborators that are residents of the country where the research was conducted or members of the community studied? If you do not have any authors from said communities, please provide an explanation for this below.

Reported on page number: Page 7

Yes, the study involved local collaborators that are residents of the country where the research was conducted. These include Chaza Akik, joint first author, and five other co-authors.

Everyone listed as an author should meet PLOS’ criteria for authorship and all individuals who meet these criteria should be included in the author byline, rather than the acknowledgements. Authorship criteria is based on the International Committee of Medical Journal Editors (ICMJE) Uniform Requirements for Manuscripts Submitted to Biomedical Journals - for further information please see here: <https://journals.plos.org/plosone/s/authorship>.

**Human subjects research (e.g. health research, medical research, cross-cultural psychology)**

Did you obtain written informed consent from a representative of the local community or region before the research took place? How did you establish who speaks for the community? Details of written informed consent obtained from study participants should be reported separately in the Methods section of your manuscript.

We did not obtain written informed consent from a member of the local community or region before the research took place. The research was based in four health facilities serving displaced Syrian and marginalised Lebanese communities, including one large tertiary hospital, and as such was not within one, geographically specific community. Our research design was guided by local collaborators who have experience of:

- designing and conducting research with displaced Syrian and marginalised Lebanese communities, including Dr Fouad M.Fouad, Co-Director of the Refugee Health Program at Global Health Institute, American University of Beirut (AUB).
- providing health services for displaced Syrian and marginalised Lebanese communities

It was reviewed by the Ministry of Public Health, and the AUB Institutional Review Board (page 7).

Details of informed consent with individual study participants are reported on pages 5-6 of the Methods section. This was done remotely using an oral script, electronically provided documents sent by text message, and a written recruitment log, following AUB COVID-19 social distancing requirements.

How did members of the local community provide input on the aims of the research investigation, its methodology, and its anticipated outcome(s)?

Members of the local community (i.e. patients using services at the study health facilities) did not provide input on the aims, methodology or anticipated outcomes of the research investigation. Local collaborators including health facility staff provided input on the aims, methodology, outcomes, and contributed to interpretation and prioritisation of findings in a stakeholder workshop to discuss preliminary findings and identify priority themes for further exploration in subsequent analysis (Methods section, page 6).

When engaging with the local community, how did you ensure that the informed consent documents and

other materials could be understood by local stakeholders?

Materials were designed by bilingual (English and Arabic) researchers based in Lebanon, experienced in conducting research with displaced Syrian and marginalised Lebanese communities, following AUB guidance for study information sheets and consent documents. English and Arabic versions were reviewed by the wider study team, which included collaborators experienced in providing health services for displaced Syrian and marginalised Lebanese communities, and by AUB IRB. Materials were piloted and refined as needed.

Potential participants were contacted twice, once for initial invitation to participate in the study, and then again separately to go through the informed consent process, so they had opportunity on two different occasions to ask questions of the same researcher. During the informed consent process, the researcher took care to check the potential participant’s understanding of materials, and prompted for questions. The researcher also took care to begin the process again with care-giver participants nominated by patients, even where they were already familiar with some of the materials, and checked that they understood that the researchers were interested in learning about their own perspectives as caregivers, rather than speaking on behalf of the patient.

Will the findings of the research be made available in an understandable format to stakeholders in the community where the study was conducted (e.g. via a presentation, summary report, copies of publications, etc.)? Please provide details of how this will be achieved.

Findings were made available to professional stakeholders (health service providers) through a stakeholder workshop, and a written summary report circulated electronically. Copies of publications will also be circulated electronically.

Findings were not made available to patient and care-giver participants because we did not judge that this was necessary use of participant's time and project resources for this study topic, design and participant group. Social distancing requirements meant that any feedback processes would need to be via telephone/virtual, and given the substantial workload experienced by patients in engaging in care to manage their conditions (as discussed in our manuscript), we did not ask them to engage in additional research related activities following their interviews.

**Non-human subjects research using specimens/ animals collected as part of the study, or those housed in archival collections. Examples include archaeology, paleontology, botany and zoology.**

Did the permission you obtained from a local authority to perform the study include an agreement on access to outputs and benefit sharing? This may include procedures to enable fair distribution of the benefits and resources arising from the research performed. Please include any details of Prior Informed Consent and Benefit Sharing Agreements obtained. These may be required by field-specific regulations, for example the Convention on Biological Diversity (CBD) and the associated Nagoya Protocol.

N/A

If the material used in your study was imported, please A) provide the year it was imported and B) indicate whether permits were obtained to import/export the materials used, C) provide details of any permits obtained. If this information is not available, please indicate this.

N/A

If you used archival specimens, please state how the material used in your study was acquired by the institute it is held in and provide details of any permits obtained for the original excavations/ sample collection. If this information is not available, please indicate this.

N/A

How was the potential cultural significance of the materials collected in your study to local communities considered in your research design? Were Indigenous peoples and/or local researchers and institutions involved with archaeological excavations / collection of specimens? If so, please provide a description of their involvement.

N/A

If your manuscript includes photographs of human remains please indicate whether authors obtained permission from descendants or affiliated cultural communities to do so.

N/A
