# Supplementary material for: Patient experiences of diabetes and hypertension care during an evolving humanitarian crisis in Lebanon: A qualitative study
Source: PLOS Glob Public Health. 2023 Dec 6;3(12):e0001383. doi: 10.1371/journal.pgph.0001383 (PMC10699589; doi:10.1371/journal.pgph.0001383)
Supplement: Attachment — Submitted filename: Response to reviewers_PGPH-D-22-01619.docx [file pgph.0001383.s005.docx]

26 May 2023

Dear Editor,

We would like to thank the three peer reviewers for their insightful and supportive reviews our manuscript ‘Patient experiences of diabetes and hypertension care during an evolving humanitarian crisis in Lebanon: a qualitative study’ [PGPH-D-22-01619].

As requested, we have:

1) Reviewed our reference list to ensure that it is complete and correct.
2) Uploaded a completed copy of PLOS’ questionnaire on inclusivity in global research as Supporting Information, and referred to this in our manuscript ( page 7, lines 194-196).
3) Sent a completed ‘Competing Interest’ statement.
4) Added a list of uploaded Supporting Information files after the References list.
5) Responded to reviewers’ suggestions as detailed below.

Responses to Reviewers’ suggestions

| Reviewer suggestions | Author response |
| --- | --- |
| Comments to the Author  Questions 1-4 (publication criteria, statistical analysis, data availability, language and presentation): no comments to address | [No comments to address] |
| Reviewer comments to the Author, section 5.  ***Reviewer #1***  *Very well conducted and written study. This is a very much needed area of research.*  *I particularly appreciated the typologies described the attention to caregivers and the “workload of demands”.* | Thank you for the positive feedback. |
| *Minor suggestions*   1) *The six recommendations are very good. I think in addition to grouping them in the conclusions, a table or figure may enhance the impact of the article.* | Thank you for the suggestion. We have added a box (Figure 1, uploaded separately from the main manuscript) highlighting a summary of the recommendations, and referred to this in the manuscript (Conclusion p20 line 624) |
| 2) *I suggest that the recommendations include specific ways to bolster support for those who do not have adequate family support as they are clearly more at risk for being in typology 3 category.* | Thank you for raising this. We have expanded recommendation two to include the need to strengthen support for patients who do not have adequate family support ( p20 line 643-4). We haven’t made recommendations on specific ways to do this as it was not explored in the research, so we are not in a position to elaborate further on the issue in this paper. |
| 3) *I understand that another article in this project that will tackle the health system, still, it would be good to add to the last recommendation the importance of strengthening the overburdened public sector and adequate regulation and oversight of the private sector during humanitarian crises in addition to the already stated recommendation of improving the coordination between the humanitarian sectors* | Thank you, we have added these suggestions. |
| ***Reviewer #2****: A joy to read, the background, context and method were clearly explained, the themes congruent with the data. This is a paper that shall influence thinking and action in this crucial shift to patient centred approaches.* | Thank you, we’re delighted to hear you enjoyed reading our paper and we hope it will be influential as predicted. |
| ***Reviewer #3****: Dear Dr. Willis,*  *What a pleasure to review your manuscript titled "Patient experiences of diabetes and hypertension care during an evolving humanitarian crisis in Lebanon: a qualitative study"; very well written, relevant and easy to follow*.  *Congratulations on the important work and excellent paper.* | Thank you for the positive feedback, very much appreciated by the study team. |
| *I have a few minor points for consideration*   1) *It would be helpful to expand a bit on the conceptual framework used for the "deductive" portion of the qualitative analysis (Page 5, Line 159). This will help identify which findings were driven by prior theory rather than inductively derived from patient experience. This could be done in writing, or maybe visually including the different phases along the continuum of care.* | Thank you for this suggestion. The majority were identified inductively and this has been clarified in the manuscript (Page 6, lines 180-181).  As background to this, this ‘patient experiences’ paper reports patient and care-giver specific perspectives, from a wider study which also included health care providers and key policy stakeholders. The conceptual framework for the overall study focuses on the health system level, and was found to be most informative in framing analysis of the health care provider and stakeholder data. The focus of the overarching conceptual framework has been clarified in the manuscript (page 6, line 171-2). |
| 2) *Was there any member checking (sharing the analyzed data or findings with the participants and asking them to confirm, refute, or provide further insight into the results) following the analyses?* | No, there was not any member (patient/care-giver) checking. As noted in our COREQ checklist response (point 28), for patients and care-givers we did not judge that this was necessary use of participant's time and project resources for this study topic, design and participant group, given that social distancing requirements meant that any feedback processes would need to be via telephone/virtual. Findings were shared with humanitarian partner country and HQ level teams, including health care providers working directly with patients, who provided feedback to confirm and give insight into results based on their experiences, and highlight key themes for further analysis. |
| 3) *Finally, while I understand that the transcripts of interviews can't be shared; it would be possible to share some data in terms of how many unique codes were derived from the transcripts, how categories these codes were merged in (you could include those as supplement) and how these categories subsequently led to the identified themes. A table or supplement with categories and themes, in addition to the narrative write-up will give readers the opportunity to review all findings in a single glance.* | Thank you for the suggestion.  Both our analysis and reporting operate at several levels, so it is unfortunately not straightforward to present a table of categories and themes which both accurately represent the analytical process, and help the reader review all the findings in a single glance.  As described in the COREQ checklist (q 25) the overall initial coding framework reflected the research priorities of the wider project, and this paper presents a subset of these results which cut across different sections and levels of the initial coding framework. Our full analysis involved several stages undertaken for different purposes (first producing preliminary findings to share at a stakeholder workshop and inform humanitarian partner programme decisions, then focusing on several themes prioritised by stakeholders in greater depth, then in-depth analysis of specific cases/case pairings, including deviant cases, some through open coding). The identification of themes was therefore iterative, and an accurate visual representation of this would demonstrate the complex iterative process, but not help the reader visualise the outcome.  We therefore felt that it would not aid the readers' understanding of the analysis process and so have not added a table/supplement.  However, in response to these comments, we have reviewed and updated the Methods to ensure the analysis process is described as fully as possible (p6 lines 175-180). |

Kind regards,

Ruth Willis (Corresponding Author), on behalf of Co-Authors
