# Supplementary material for: Patient experiences of diabetes and hypertension care during an evolving humanitarian crisis in Lebanon: A qualitative study
Source: PLOS Glob Public Health. 2023 Dec 6;3(12):e0001383. doi: 10.1371/journal.pgph.0001383 (PMC10699589; doi:10.1371/journal.pgph.0001383)
Supplement: Attachment — Submitted filename: Note re response to reviewers.docx [file pgph.0001383.s006.docx]

Please note: the response to Editor’s comments is included in the Cover Letter. There were no comments from reviewers to response to in this revision, but the submission system required a document to be uploaded in this ‘Item Type’ in order to proceed with the resubmission.

Many thanks, Ruth Willis
